# Peroxisome Proliferator-Activated Receptor **γ** Regulates the Expression of Lipid Phosphate Phosphohydrolase 1 in Human Vascular Endothelial Cells

**DOI:** 10.1155/2014/740121

**Published:** 2014-05-12

**Authors:** Yazi Huang, Beilei Zhao, Yahan Liu, Nanping Wang

**Affiliations:** Institute of Cardiovascular Science, Peking University Health Science Center, Beijing 100191, China

## Abstract

Lipid phosphate phosphohydrolase 1 (LPP1), a membrane ectophosphohydrolase regulating the availability of bioactive lipid phosphates, plays important roles in cellular signaling and physiological processes such as angiogenesis and endothelial migration. However, the regulated expression of LPP1 remains largely unknown. Here, we aimed to examine a role of peroxisome proliferator-activated receptor **γ** (PPAR**γ**) in the transcriptional control of *LPP1* gene expression. In human umbilical vein endothelial cells (HUVECs), quantitative reverse transcriptase polymerase chain reaction (qRT-PCR) demonstrated that activation of PPAR**γ** increased the mRNA level of LPP1. Chromatin immunoprecipitation assay showed that PPAR**γ** binds to the putative PPAR-responsive elements (PPREs) within the 5′-flanking region of the human *LPP1* gene. Genomic fragment containing 1.7-kilobase of the promoter region was cloned by using PCR. The luciferase reporter assays demonstrated that overexpression of PPAR**γ** and rosiglitazone, a specific ligand for PPAR**γ**, could significantly upregulate the reporter activity. However, site-directed mutagenesis of the PPRE motif abolished the induction. In conclusion, our results demonstrated that PPAR**γ** transcriptionally activated the expression of *LPP1* gene in ECs, suggesting a potential role of PPAR**γ** in the metabolism of phospholipids.

## 1. Introduction

Lipid phosphate phosphohydrolases (LPPs), also known as phosphatidate phosphohydrolase-2 (PAP-2), are the Mg^2+^-independent and N-ethylmaleimide-insensitive N-glycosylated integral membrane ectophosphohydrolase [1, 2]. LPPs catalyze the dephosphorylation of a range of lipid phosphates, such as lysophosphatidic acid (LPA) and sphingosine 1-phosphate (S1P) [3, 4]. Extracellular LPA and S1P bind to the G-protein-coupled receptors (GPCRs) and exert a number of pathophysiological actions, such as angiogenesis, platelet activation, inflammation, smooth muscle cells (SMCs) proliferation and migration, and cardiovascular remodeling [4, 5]. LPPs hydrolyze these lipid phosphates to terminate their signaling actions or generate new signaling molecules [6]. Three isoforms of LPPs (LPP1, LPP2, and LPP3) have been found [7]. LPP1 negatively regulates lysophospholipid signalings by degrading the bioactive lysophospholipids released from platelets and modulates their effects on the cell proliferation, migration, inflammation, coagulation, and wound healing [5, 6]. The activity of LPP1 is mainly regulated through de novo expression rather than posttranslational modification such as phosphorylation. Expression of* LPP1* was induced by androgens in human prostatic adenocarcinoma cells and decreased in ovarian cancers [8, 9]. However, transcriptional mechanism underlying the regulation expression of the* LPP1* remains largely unclear.

Peroxisome proliferator-activated receptors (PPARs) are a family of ligand-activated nuclear receptors and transcription factors [10]. Among three PPAR isoforms (*α*, *β*/*δ*, and *γ*), PPAR*γ* is predominantly expressed in adipose tissue and also in vasculature including vascular smooth muscle cells (VSMCs) and endothelial cells (ECs) [11, 12]. PPAR*γ* forms a heterodimer with RXR and binds to the PPAR response elements (PPREs) in the promoter region of target genes [13]. When activated by various natural and synthetic ligands such as prostaglandin metabolite 15d-PGJ2 [14] and the insulin sensitizer rosiglitazone [15], PPAR*γ* transactivates the gene expression and regulates adipogenesis [16] and insulin response [17]. In addition, PPAR*γ* possesses antiatherogenic and anti-inflammatory actions in ECs [18, 19]. Therefore, we attempted to examine a role of PPAR*γ* in the regulation of* LPP1* gene expression in ECs.

## 2. Materials and Methods

### 2.1. Cell Culture and Reagents

Human umbilical vein endothelial cells (HUVECs) were cultured as previously described [20]. Bovine aortic endothelial cells (BAECs) were harvested from bovine aorta and maintained in DMEM with 10% FBS [21]. Rosiglitazone, GW501516, and GW9662 were obtained from Cayman Chemical. Polyclonal rabbit anti-PPAR*γ* and rabbit IgG were from Santa Cruz Biotechnology. Luciferase assay reagent, MMLV reverse transcriptase, Taq polymerase, restriction enzymes (XhoI, NheI), and DNA ligase were from Promega Corporation. Lipofectamine 2000 and Trizol reagent were obtained from Invitrogen. The QuikChange site-directed mutagenesis kit was from Stratagene Corporation.

### 2.2. Adenoviral Infection

Cells were infected with adenoviruses encoding the wild type human PPAR*α*, *β*/*δ*, or *γ*1 (Ad-WT-PPAR*α* or Ad-WT-PPAR*β*/*δ*, Ad-WT-PPAR*γ*) together with Ad-tTA, which encodes a tetracycline-responsive transactivator. These viral constructs were previously described and used at 50 multiplicities of infection [22, 23]. Infected cells were maintained in the presence or absence of tetracycline (0.1 *μ*g/mL, a tet-off expression) for 48 hours as described [20].

### 2.3. RNA Extraction and Real-Time Quantitative RT-PCR (qRT-PCR)

Total RNA was extracted with Trizol reagent and reverse transcribed into cDNA with M-MLV reverse transcriptase with oligo-dT as a primer. Real-time PCR was performed with SYBR-green dye and Taq polymerase in the DNA Engine Opticon real-time system (Bio-Rad Laboratories Inc.). GAPDH was used as an internal control. The primer sequences are: LPP1 5′-TCAACTGCAGCGATGGTTAC (forward), 5′-GCCCACATAAATGGATACGG (reverse); GAPDH 5′-ACCACAGTCCATGCCATCAC (forward), 5′-TCCACCACCCTGTTGCTGTA (reverse).

### 2.4. Plasmids, Mutation, Transfection, and Reporter Assay

The genomic fragment containing −1921 to −221 bp upstream of the transcription start site of human* LPP1* gene was PCR amplified from human genomic DNA with the primers (5′-CTTGATAGTACAACAGGGTCA and 5′-TCAGGTGGTCTCCGAACT) with flanking sites of NheI and XhoI. The amplified product was subcloned into the pGL3-basic luciferase vector to generate the pGL3/LPP1-luc. The Quickchange site-directed mutagenesis kit was used to generate the pGL3/mLPP1-luc by disruption of the putative PPRE site (from −624 to −611 bp) with the use of the mutagenic primers: 5′-GAGGGATTCTGGCTAAAGGCG(A)GT(G)TCCC(AA) GGT(G)CTTCTACAAC and 5′-GTTGTAGAAGA(C)CCGG(TT) GAA(C)CC(T)GCCTTTAGCCAGAATCCCTC. The plasmids were transfected together with the pRSV-*β*-gal plasmid into BAECs by using Lipofectamine 2000. Luciferase activity was measured with the luciferase assay kit and normalized with *β*-galactosidase activity.

### 2.5. Chromatin Immunoprecipitation

HUVECs were infected with Ad-WT-PPAR*γ* and, 48 h later, cross-linked with 1% formaldehyde. The sheared chromatin DNAs were immunoreacted with 2 *μ*g anti-PPAR*γ* antibody (or IgG as negative control) and precipitated with protein A/G sepharose beads. The eluted immunoprecipitates were digested with proteinase K. DNA was amplified by qPCR with the primers flanking the putative PPREs. The primers for ChIP assay were shown in Table 1.

### 2.6. Statistical Analysis

Data are expressed as mean ± SEM from at least three independent experiments. Statistical analyses were performed by using one-way ANOVA. The *P* values less than 0.05 were considered statistically significant.

## 3. Results

### 3.1. PPREs Are Recurrent Motifs in the 5′-Flanking Region of Human* LPP1* Gene

**Figure 1:**
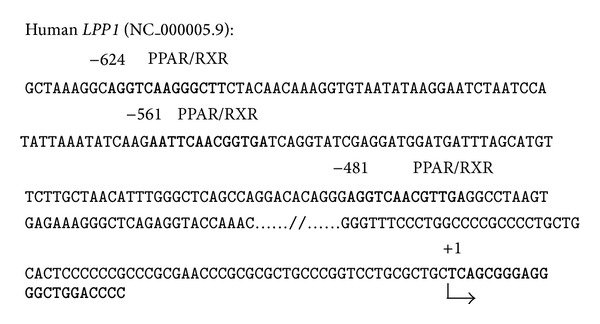
Putative PPAR-responsive elements (PPREs) in 5′-flanking region of the human* LPP1* gene. Three putative PPREs were located in 5′-flanking region of the human* LPP1* gene (NC_000005.9). Nucleotide numbers are relative to the transcription start site (+1, arrow).

We analyzed the human* LPP1* 5′-flanking (NC_000005.9) using MatInspector (http://www.genomatix.de/) and identified three putative PPRE motifs, respectively, locating at −418 bp (AGGTCAACGTTGA), −548 bp (AATTCAACGGTGA), and −611 bp (AGGTCAAGGGCTT) upstream of the transcriptional start site of human* LPP1* gene (Figure 1).

### 3.2. PPAR*γ* Upregulates* LPP1* Gene Expression in ECs

**Figure 2:**
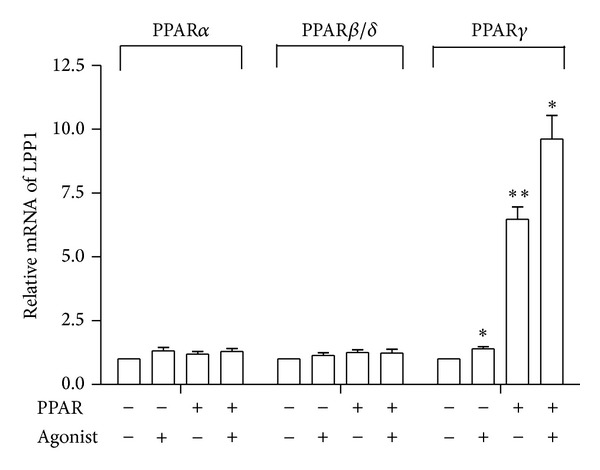
PPAR*γ* increases* LPP1* expression in mRNA levels. Confluence HUVECs were coinfected with adenoviruses expressing different isoforms of human PPARs together with Ad-tTA. Cells were maintained in the presence or absence of tetracycline for 24 h and then treated with the selective agonists (fenofibrate, GW501516, or rosiglitazone) or vehicle (DMSO). LPP1 mRNA levels were normalized to GAPDH and expressed as mean ± SEM of three independent experiments. *P < 0.05; **P < 0.01 versus control.

To examine whether PPAR*γ* regulates* LPP1*, we infected HUVECs with Ad-WT-PPAR*γ* together with Ad-tTA in the presence or absence of tetracycline (0.1 *μ*g/mL) for 24 h. Then, cells were treated with the PPAR*γ* ligand rosiglitazone (5 *μ*M) for 24 h. The qRT-PCR results showed that mRNA level of LPP1 was significantly induced in ECs activated with rosiglitazone or overexpressing PPAR*γ*. Rosiglitazone further augmented the induction by PPAR*γ* overexpression (Figure 2).

To investigate whether other two isoforms of PPARs also have similar effects, we also infected the ECs with Ad-WT-PPAR*α* or Ad-WT-PPAR*δ* and treated with their specific agonists fenofibrate (5 *μ*M) and GW501516 (1 *μ*M) for 24 h. LPP1 mRNA level were not affected by neither PPAR*α* nor PPAR*β*/*δ* overexpression. Similarly, PPAR*α* and PPAR*β*/*δ* agonists had no effect on* LPP1* expression (Figure 2).

**Figure 3:**
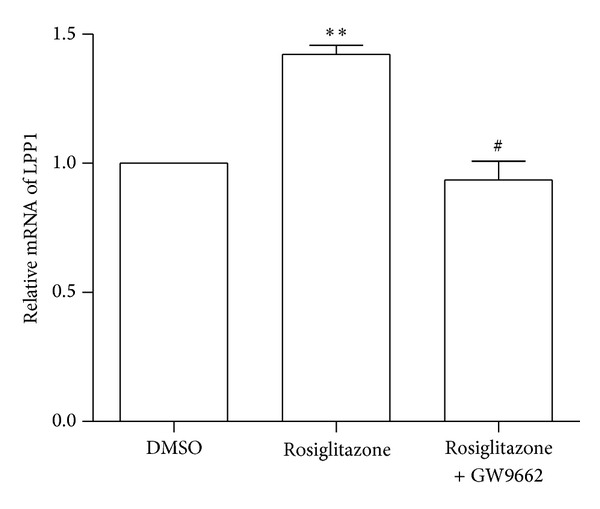
PPAR*γ* antagonist attenuates the induction of LPP1 by rosiglitazone. HUVECs were pretreated with GW9662 (20 *μ*M) before the exposure to rosiglitazone. Total RNA were extracted 36 h later and assessed by using qRT-PCR for LPP1 mRNA expression. **P < 0.01, versus control (DMSO); ^#^ *P* < 0.05, versus rosiglitazone.

In order to examine whether the effect of rosiglitazone was specific for PPAR*γ*, we used GW9662, a selective antagonist, to pretreat ECs before the exposure. As shown in Figure 3, PPAR*γ* antagonism significantly attenuated the LPP1 induction by rosiglitazone. Taken together, these results indicated that* LPP1* gene was induced by PPAR*γ* activation.

### 3.3. PPAR*γ* Binds to the PPRE in the Promoter of Human* LPP1* Gene

**Figure 4:**
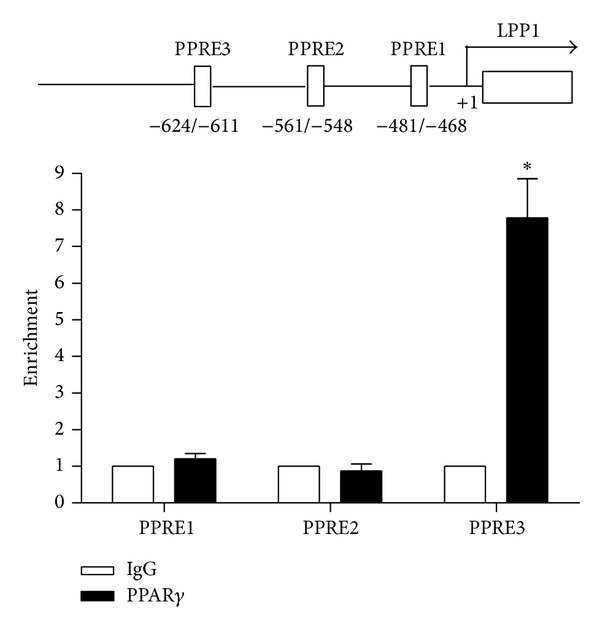
PPAR*γ* binds to the PPRE within the 5′-flanking region of LPP1. HUVECs were coinfected with Ad-WT-PPAR*γ* and Ad-tTA for 48 h. ChIP assays were performed with the antibodies against PPAR*γ* or IgG as a negative control. Immunoprecipitated DNA fragments were detected by qPCR with the use of specific primers spanning the DNA segments containing the predicted PPREs of LPP1 promoter. Data were mean ± SEM of three independent experiments. *P < 0.05 versus control.

Sequence analysis of the 5′-flanking region of human* LPP1* gene revealed three putative PPRE motifs. To examine whether PPAR*γ* binds to these regions, ChIP assay was performed with the anti-PPAR*γ* antibody and IgG as control. The results showed that PPAR*γ* could bind to the PPRE located at −624/−611 bp upstream of the human* LPP1* gene, while the two proximal sites at −481/−468 and −561/−548 had no binding (Figure 4). This result suggested that the PPRE at −624/−611 might mediate the induction by PPAR*γ*.

### 3.4. PPAR*γ* Increases the Promoter Activity of Human* LPP1* Gene via Binding to PPRE3

**Figure 5:**
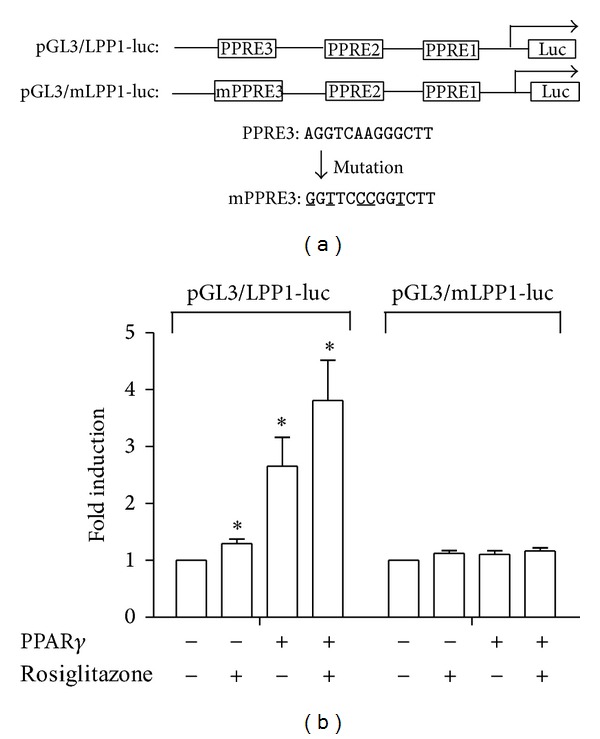
PPAR*γ* increases the promoter activity of LPP1 via binding to PPRE. (a) The pGL3/LPP1-luc and pGL3/mLPP1-luc plasmids with the 1.7 kb of 5′-flanking region of the human* LPP1* gene with 3 putative PPREs and the mutations depicted. (b) BAECs were transfected with pGL3/LPP1-luc or pGL3/mLPP1-luc plasmids together with *β*-gal plasmids. Data were fold induction of luciferase activity and expressed as mean ± SEM of four independent experiments. *P < 0.05; **P < 0.01 versus control.

To further examine whether PPAR*γ* promotes the* LPP1* gene transcription activity, we constructed pGL3/LPP1-luc reporter driven by the fragment containing three putative PPREs (Figure 5(a)). BAECs were transfected with pGL3/LPP1-luc plasmid. Luciferase assay showed that the reporter activity was increased by rosiglitazone or overexpression of PPAR*γ* (Figure 5(b)). However, site-directed mutagenesis of the PPRE at −624/−611 abolished the induction by PPAR*γ*, indicating that the PPRE at −624/−611 is the cis-element mediating the PPAR*γ* transactivation.

## 4. Discussion 

In the present study, we have demonstrated a transcriptional mechanism regulating the expression of* LPP1* gene. We provided novel evidence that PPAR*γ* and its specific agonist rosiglitazone positively regulate the transcription of* LPP1* gene in ECs. We also identified the PPRE motif within the regulatory region of the* LPP1* gene that mediates the action of PPAR*γ*.

Elucidation of the transcriptional regulation of* LPP1* is of physiological importance because* LPP1* is a key enzyme responsible for the catabolism of LPA and S1P which are the bioactive lipid phosphates [5]. Therefore, given the mass and extensive distribution of ECs, the induction of* LPP1* expression may lead to a profound decrease in the concentrations of LPA and S1P, both in circulation and in the tissues, and exert important biological effects. Although PPAR*γ* has its major roles in the regulation of adipogenesis and insulin sensitivity, it also has a variety of protective effects on cardiovascular functions ranging from atherogenesis to blood pressure regulation [24, 25]. The finding that PPAR*γ* induces* LPP1* expression in ECs may provide new insights into the molecular mechanisms underlying the cardiovascular effects of PPAR*γ* modulation.

LPP1 is an ectoenzyme with its active sites located on the outer surface of plasma membrane. It degrades extracellular LPA to monoacylglycerol, which can be taken into cells to produce intracellular LPA by acylglycerol kinase. Intriguingly, intracellular LPA was known to be an agonist for PPAR*γ* [26, 27]. Thus, it is postulated that the regulation of LPP1 by PPAR*γ* may act as positive loop for extracellular LPA degradation. In human atherosclerotic lesions, LPA was accumulated in the lipid core of the plaques and promoted the development of atherosclerosis in vivo [28, 29]. Lipoprotein-derived LPA promoted the atherosclerosis in animal models [30]. In contrast, PPAR*γ* agonists thiazolidinediones (TZD) reduced atherosclerotic plaques both in diabetic patients and in animal models [31]. Therefore, whether the induction of LPP1 and ensuing decrease in LPA contribute to the antiatherosclerotic effect of PPAR*γ* remains to be investigated. In addition, the effects of PPAR*γ* activation on the protein level, enzymatic activity of LPP1, and extracellular levels of LPA and S1P need to be examined in the future.

## Figures and Tables

**Table 1:** The sequences of the primers for ChIP assay.

hLPP1 PPRE1	5′-AGGTGACGGTGGATGGAA-3′
	5′-CCTTTGTTGTAGAAGCCCTT-3′

hLPP1 PPRE2	5′-AGGGCTTCTACAACAAAGG-3′
	5′-ATCATCCATCCTCGATACCT-3′

hLPP1 PPRE3	5′-CGAGGATGGATGATTTAGCA-3′
	5′-GAGCCCTTTCTCACTTAGG-3′
